# Evaluation of Acephate Metabolites Generated by Zebrafish ( *Danio rerio* ) Using LC–HRMS and Metabolomics Approach

**DOI:** 10.1002/jat.4988

**Published:** 2025-11-17

**Authors:** Mariana Laiz Silva de Lima, Lucas Rodrigues Cunha, Karina Taciana Santos Rubio, Maria Elvira Poleti Martucci

**Affiliations:** ^1^ Postgraduate Program in Environmental Engineering – ProAmb Federal University of Ouro Preto Ouro Preto Minas Gerais Brazil; ^2^ Department of Pharmacy, School of Pharmacy Federal University of Ouro Preto Ouro Preto Minas Gerais Brazil

**Keywords:** acephate, *Danio rerio*, LC–HRMS, metabolomics, pesticides

## Abstract

Acephate is an organophosphate insecticide widely used. This pesticide can be metabolized into methamidophos, a molecule highly toxic. Both have demonstrated the ability to promote serious toxic effects on nontarget organisms and have been identified in surface waters around the world. Therefore, it is important to understand their behavior in the environment and in living organisms. Zebrafish are considered an excellent animal model for studying the toxicity of xenobiotics present in water. This work aimed to identify acephate metabolites formed in zebrafish and excreted into tank water using liquid chromatography coupled to a high‐resolution spectrometer (LC–HRMS) to obtain biomarkers of exposure in aquatic environments. For this purpose, three groups were established: treatment, negative, and stability. After exposure, water samples from the tanks were collected for analysis by LC–HRMS. The experiments allowed the putative identification of three acephate metabolites that can be used to monitor exposure to this pesticide with a high degree of confidence. The approach used to identify these exposure markers shows promise since water is a cleaner and easier‐to‐obtain matrix when compared to biological matrices. To the best of our knowledge, this is the first study on the identification of acephate metabolites in zebrafish.

## Abstract 2

Acephate is an organophosphate insecticide that can be metabolized into methamidophos. Both may promote toxic effects and have been identified in surface waters. Therefore, it is important to understand their behavior in the environment and in living organisms. This work aimed to obtain biomarkers of acephate exposure by identifying the metabolites formed in zebrafish and excreted into tank water. Analysis of water samples by LC–HRMS allowed the identification of three acephate metabolites that can be used to monitor exposure.

## 1 Introduction

Acephate (O,S‐dimethyl‐acetyl‐phosphoramidothioate) is an organophosphate insecticide widely used to control sucking, chewing, aphid, and thrips pests in both agriculture and domestic environments (Kumar et al. 2015; Lewis et al. 2016). It can be degraded into methamidophos, a metabolite with a higher degree of toxicity (Class IB—highly hazardous) (Araoud et al. 2016; Fernandes Mendonça Mota et al. 2023; WHO 2019).

Like other organophosphates, acephate and methamidophos are capable of binding to the enzyme acetylcholinesterase (AChE) present in the nervous system and blood, promoting its inhibition and consequent accumulation of acetylcholine in synaptic clefts (Tsai and Lein 2021). Although it is classified as a “moderately hazardous” pesticide (Class II) (WHO 2019), different toxic effects related to acephate exposure have been observed in target and nontarget organisms (Fernandes Mendonça Mota et al. 2023). Among such effects: damage to the reproductive system (Sampaio et al. 2020; Sasaki et al. 2023), endocrine disruption (Blanco‐Munoz et al. 2010), neurotoxicity (Baldi et al. 2001), and hepatotoxicity (Chang et al. 2009; Hou et al. 2015).

As a result of these effects, the use of acephate has been restricted in several countries (Chowdhary et al. 2014). Despite this, residues of acephate and methamidophos have been detected in water samples around the world. In the Guadalquivir River basin of Spain, residues of acephate and methamidophos were detected at concentrations of 470 and 90 ng/L, respectively (Espigares et al. 1997). Between 2016 and 2017, in China, average acephate concentrations of 1.67 μg/L were detected in the Yangtze River and 1.31 μg/L in the Huangpu River (Sun et al. 2018). In Chile, a study that evaluated pesticides in the Cachapoal River Basin detected acephate at concentrations of 5.2 and 2.2 μg/L, during the spring and autumn seasons, respectively (Climent et al. 2019). Recently, in the Periyar River, India, the average concentrations of acephate detected during the dry season (summer) and monsoon (rainy) varied between 0.01 and 0.4 μg/L and 0.025 and 0.1 μg/L, respectively (Gayathri et al. 2021). In Brazil, between 2018 and 2020, concentrations ranging from 0.01 to 100 μg/L of methamidophos, a byproduct of the pesticide acephate, were detected in surface waters (Brovini et al. 2023).

Considering the frequent detection of acephate and methamidophos in waters, understanding their metabolism in fish and using such metabolites to monitor and mitigate the effects of exposure to such substances in the aquatic environment is extremely important. To this end, the metabolomics approach has been successfully used to assess exposure to different xenobiotics (Matos et al. 2019). The zebrafish ( *D. rerio* ) has become an excellent animal model for evaluating toxicity, mainly because it is an organism with anatomy, physiology, and development similar to higher vertebrates, for providing quick and cheap tests and for easily absorbing substances present in water (de Souza Anselmo et al. 2018; Otte et al. 2017).

Thus, the aim of this work was to evaluate the metabolites formed from acephate by zebrafish in water using liquid chromatography coupled to a high‐resolution mass spectrometer (LC–HRMS). The identification of such molecules can highlight possible biomarkers to be used in monitoring exposure to acephate in aquatic environments.

## 2 Materials and Methods

### 2.1 Chemicals and Reagents

Acephate (purity ≥ 98%), methamidophos (purity ≥ 98%) and proxyphylline (internal standard, purity ≥ 98%) were acquired from Sigma–Aldrich (Missouri, USA). The HPLC grade solvents (formic acid, ammonium formate, and methanol) were obtained from J. T. Backer (Pennsylvania, USA). The enzyme β‐glucuronidase from *Escherichia coli* was supplied by Roche (Mannheim, Germany). Spe‐ed Octadecyl C18/18% (200 mg/6 mL) cartridges were acquired from Applied Separations (Allentown, USA). Chlorine neutralizer (AquaSafe) was purchased from Tetra (Blacksburg, VA, USA). The colorimetric tests to evaluate dissolved O_2_, ammonia, and nitrite were supplied by Alcon (Camboriú, Brazil).

### 2.2 Zebrafish Maintenance

Adult zebrafish ( *D. rerio* ), from both sexes, measuring around 2 cm were obtained from a commercial supplier (Lindoia, Minas Gerais state) and acclimated to water tank conditions throughout 14 days before the assay. The fish were kept in aerated tanks containing 4.0 L of dechlorinated tap water at 26°C ± 2°C. The tanks were kept at a controlled period of light (14 h) and dark (10 h) and were fed daily using commercial ration (Alcon, Camboriú, Brazil). The water tanks were weekly monitored for ammonia, nitrite, dissolved O_2_, pH, and temperature. Prior to the execution, the study was submitted and approved by the Ethics Committee on the Use of Animals of the Federal University of Ouro Preto (Protocol Number 7061120319).

### 2.3 Biotransformation Assay With Acephate

Adult fish (*n* = 60) were randomly distributed in three treatment tanks (TT) containing acephate at 200 μg/L. Three additional tanks (stability tanks [STs]) with acephate at 200 μg/L and free of fish were prepared. A single tank with clean tap water and with 20 fish was used as a negative tank (NT). During the assay, two samples containing 10 mL were collected from each tank at 0, 1, 3, 5, 8, 10, 24, 48, 72, 96, 120, 144, and 168 h. The concentration of 200 μg/L was chosen as it allowed the identification of metabolites generated by zebrafish biotransformation.

### 2.4 Sample Preparation

Initially, the SPE cartridges were conditioned with methanol (3 mL) and water (3 mL). Before sample loading onto cartridges, a 10 μL aliquot of each sample was put in a vial, followed by the addition of the proxyphylline at a concentration of 50 μg/L. After that, 80 μL of *β*‐glucuronidase enzyme was added to each sample. The samples were submitted to hydrolysis in a water bath kept at 50°C for 1 h. Subsequently, the samples were applied to the cartridges, and the analytes were eluted by the addition of 3 mL of methanol. The obtained sample was dried with nitrogen. The dried residue was reconstituted in 60 μL of a solution composed of water: methanol (7:3) with 0.1% formic acid. Then, the reconstituted sample was pooled in the 10 μL aliquot and analyzed by LC–HRMS.

### 2.5 Liquid Chromatography–High‐Resolution Mass Spectrometry

The LC system used was a Dionex UltiMate 3000 UHPLC system, and the HRMS equipment was a QExactive Plus Orbitrap mass spectrometer (MS) (Thermo Fisher Scientific, Bremen, Germany), equipped with an electrospray ionization (ESI) source. Previously, the calibration of the MS in positive mode was performed with the manufacturer's calibration solutions (Thermo Fisher Scientific, Bremen, Germany) to ensure mass accuracies below 6 ppm.

The samples were analyzed using a C18 column (50 × 2.1 mm × 1.7 μm, Syncronis‐Thermo) kept at 40°C. The mobile phases were constituted by (A) water with 5‐mM ammonium formate (B) methanol, both containing 0.1% formic acid. The elution profile was: 0 min, 2% B; 3 min, 10% B; 4 min, 25% B; 7 min, 100% B; 8 min, 100%; 8.5 min, 2%; and 10.5 min, 2% B. Each sample was injected in a volume of 5 μL. The HRMS system was operated in positive mode ionization with the following parameters: spray voltage 2.89 kV, capillary temperature 380°C, S‐lens radiofrequency level 80 (arbitrary units) and auxiliary gas heater temperature was 380°C. Nitrogen sheath and auxiliary gas flow rates were set to 60 and 20 (arbitrary units), respectively. The full‐MS scan mode was acquired with the following parameters: resolution 70,000 (arbitrary unit), mass range 50–750 *m*/*z,* automatic gain control (AGC) 1e6 (arbitrary unit) and maximum injection time (IT) 50 ms. For the full‐MS/DDA‐MS2 acquisition mode, the parameters were set as follows: resolution 35,000 (arbitrary unit), mass range 50–750 *m*/*z,* AGC 1e5 (arbitrary unit) and maximum (IT) 50 ms, isolation window 2.0 *m*/*z*, loop count 10, top N 10 and (N) CE 10, 20, and 30 eV.

### 2.6 Analytical Method Validation

The analytical method was validated for acephate and its main metabolite, methamidophos, according to US EPA (USEPA, 2016) and INMETRO (INMETRO, 2020) guidelines. Therefore, the parameters evaluated were specificity, linearity, limit of quantitation (LOQ), repeatability, recovery, and carryover.

Specificity was verified by an analysis of six blank samples followed by the evaluation of interference presence at the retention time of each analyte. The coefficient of determination (*R* ^2^) from the linear regression was used to evaluate the linearity. Therefore, six concentration levels (1, 5, 20, 50, 100, and 150 μg/L) of both analytes were injected in triplicates allowing the analytical curve construction. Then, the LOQ was assigned based on the peak area with a signal‐to‐noise ratio (S/N) greater than 10, which allowed the detection of acephate and methamidophos at the lowest concentration.

Both repeatability and recovery were evaluated at 1, 50, and 150 ng/mL using five samples each. The repeatability suitability was evaluated by the determination of the relative standard deviation (RSD) of peak areas. Regarding recovery, the adding of the analytes was done after the extraction procedure, and the calculation of recovery (%) was carried out in relation to repeatability samples and according to the Equation (1)

(1) $$\text{Recovery}\;\left(\%\right)=\left(A_{\text{sample spiked after}}–A_{\text{sample spiked before}}\right)\times 100/A_{\text{sample spiked after}}$$

The carryover effect was evaluated by analysis of a blank sample analyzed before and after a sample spiked at 150 ng/mL.

### 2.7 Detection of Acephate Metabolites

The acephate metabolites annotation was based on the reported literature (Chukwudebe et al. 1984), (Singh et al. 2020), (Wu et al. 2023), (Lin et al. 2022), (Ren et al. 2020) and their exact mass calculations. Subsequently, the molecular formulas were proposed by Thermo Xcalibur software (version 3.0.63; Thermo Fisher Scientific, Bremen, Germany) having as criteria the theoretical isotope standard and lower mass error.

After that, the exact mass of each precursor ion was investigated in full‐MS scan mode followed by assessment of the MS/MS spectra generated by full‐MS/DDA‐MS2 to obtain the product ions.

### 2.8 Statistical Analysis for Metabolomics

The water aliquots collected in the time range of 0–168 h from the tanks treated with acephate (TT) and the NT were analyzed by LC–MS. The data acquired were analyzed using the freely available MS‐DIAL program version 4.19 on the RIKEN PRIMe website (Tsugawa et al. 2015). First, raw MS files were converted into ABF format by the free Reifycs file converter and the ABF files were loaded into MS‐DIAL with the following conditions: retention time range = 0.2–10 min; mass range = 50–750 Da; mass accuracy tolerance = 0.001 Da for MS1 and 0.025 Da for MS2; minimum peak height = 1000; mass slice width = 0.1 Da; smoothing method = linear weighted moving average; smoothing level = 3 scan; minimum peak width = 5 scan; sigma window value = 0.6; MS/MS abundance cutoff = 10 amplitude; exclude after precursor ion; keep the isotopic ions until 3 Da.

The target acephate metabolites were identified using an in‐house database containing the main metabolites and their respective exact mass. The applied parameters were: retention time tolerance = 10 min; accurate mass tolerance = 0.01 Da; identification score cutoff = 0.9%; relative abundance spectrum cutoff = 0.3%; only report the top hit. Additionally, the adducts [M + H]^+^ and [M + Na]^+^. Then, the applied alignment parameters were: retention time tolerance = 0.05 min; MS1 tolerance = 0.015 Da; retention time factor = 0.5; MS1 factor = 0.5; peak count filter = 5%; and N% detected in at least one group = 10%; gap filling by compulsion.

Afterward, the data set was analyzed by multivariate statistical analysis (MSA). First, the unlikely variables were removed by auto scaling and the quad root transform method. A principal component analysis (PCA) was carried out to show the samples clustering. An orthogonal partial least squares‐discriminant analysis (OPLS‐DA) was performed to highlight the important variables for that differential clustering. The quality of the OPLS‐DA model was ensured by the *Q* ^2^ value greater than 0.3, which was provided by the cross‐validation.

The score plots from the PCA and OPLS‐DA models were used to assess the clustering samples. Besides, OPLS‐DA yielded the variables important to the projection (VIPs) and values of VIP greater than 1 indicate the most important variables for discriminating the clusters. Finally, the statistical significance was assessed by *p* value < 0.05 and a fold change threshold > 2 (Costa et al. 2023).

## 3 Results

### 3.1 Analytical Method Validation

The analytical method was validated to guarantee a reliable evaluation of acephate biotransformation, mainly detection and quantitation of acephate and methamidophos in water samples. In this way, the specificity of the method showed that both analytes could be detected as no interference impaired their detection. Also, the analytical curve provided *R* ^2^ of 0.9914 and *R* ^2^ of 0.9829 for acephate and methamidophos, respectively, ensuring satisfactory linearity. In addition, the LOQ was determined as the lowest level of the analytical curve (1 ng/mL) for both analytes since it showed an S/N > 10.

Regarding repeatability, the three concentrations evaluated were suitable, showing RSD < 20%. The recovery for both analytes was also satisfactory, as it was 11.5%, 6.7%, and 99.8% for acephate at 1, 50, and 150 ng/mL, respectively, and it was 12.2%, 12.5%, and 98.4% for methamidophos at 1, 50, and 150 ng/mL, respectively. Moreover, this analytical method showed no carryover suggesting that there is no risk of contamination between the injected samples.

### 3.2 Acephate Metabolization

The acephate metabolism profile was evaluated by periodic collection of water samples during 168 h. These samples were prepared and analyzed by LC–MS. Then, we proceeded to a search in full‐MS scan mode (Figure S1) of the acephate precursor ion at *m*/*z* 184.01917 and methamidophos precursor ion at *m*/*z* 142.00861, which shows acephate metabolism and degradation. The MS/MS spectrum enabled us to evaluate the characteristic product ions of acephate, ensuring its identity: *m*/*z* 157.00224, *m*/*z* 142.99274, *m*/*z* 125.02119, and *m*/*z* 86.09697. In the same way, the MS/MS spectrum could prove the methamidophos identity by the detection of the product ions at *m*/*z* 128.97713 and at *m*/*z* 113.00007.

**FIGURE 1:**
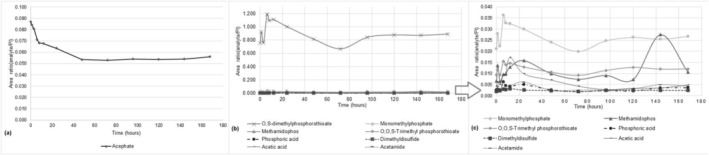
Behavior of acephate metabolism: the biotransformation ratio and the decrease in acephate in 168 h (A). Acephate metabolites formed by zebrafish ( *Danio rerio* ) in 168 h and detected in collected water samples (B). Enlargement of the observed metabolites in B to view details (C).

**FIGURE 2:**
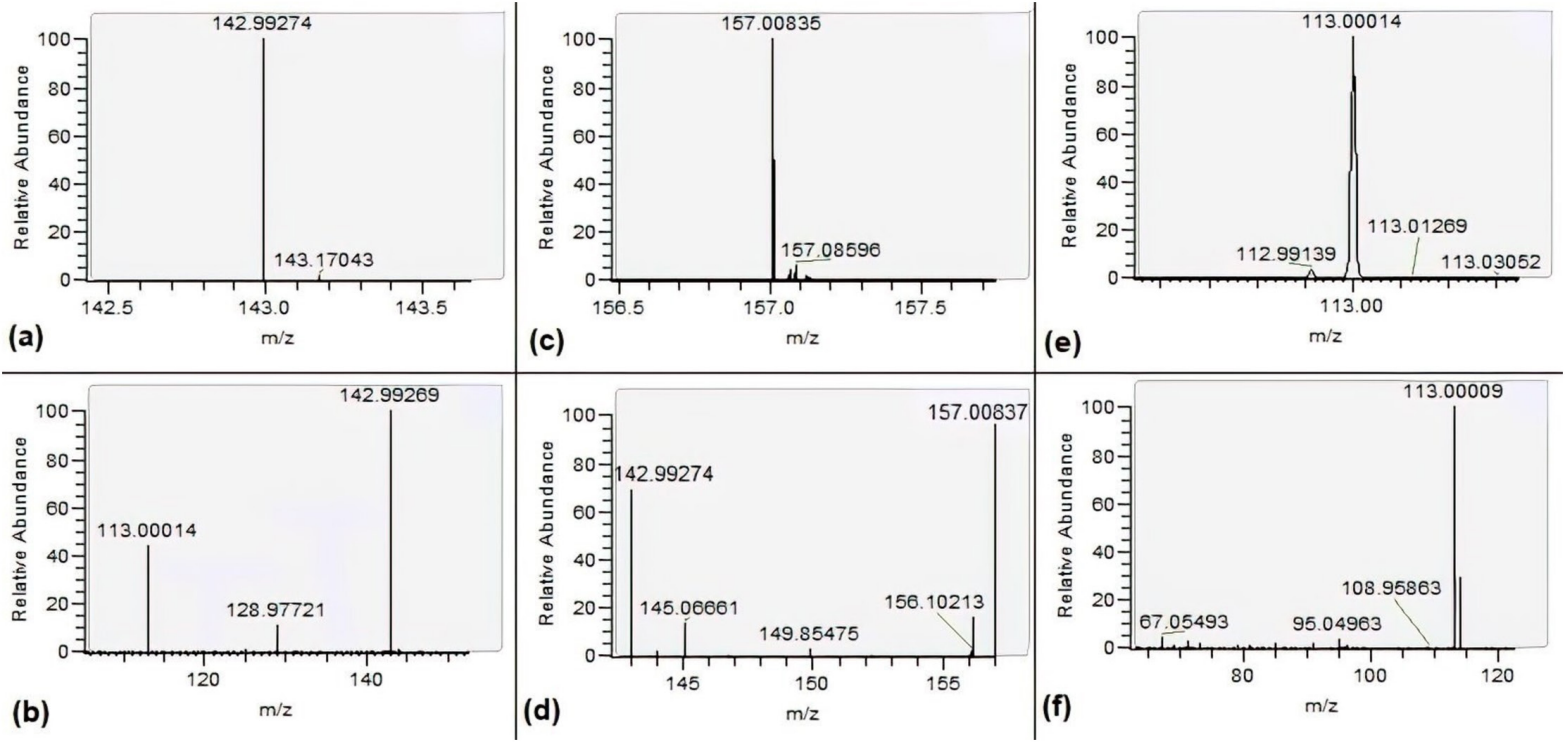
Mass spectrum in full‐scan for the metabolites O,S‐dimethylphosphorothioate (A), O,O,S‐trimethyl phosphorothioate (C), and monomethylphosphate (E), MS–MS spectrum for the O,S‐dimethylphosphorothioate (B), O,O,S‐trimethyl phosphorothioate (D), and monomethylphosphate (F) identified in the collected water samples.

**FIGURE 3:**
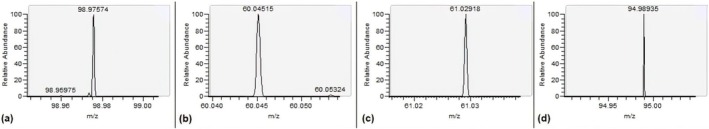
Mass spectrum in full scan for the metabolites phosphoric acid (A), acetamide (B), acetic acid (C), and dimethyldisulfide (D) identified in the water tank.

Afterward, the acephate concentration showed a trend to decrease in the first 48 h (Figure 1A). Interestingly, methamidophos was detected after 1 h, mainly at 24 h, and with the highest generation at 144 h and a remarkable decrease at 168 h (Figure 1C). Considering this methamidophos generation profile, we proceeded to a more extensive search to detect other metabolites capable of pointing out acephate exposure (Figure S2). The full‐MS spectra were used to evaluate the presence of these metabolites (Figure 2A,C,E and Figure 3). Additionally, the MS/MS spectra were evaluated to confirm the identities of these putative metabolites (Figure 2B,D,F). According to molecular formulas and the corresponding precursor and product ions (Table 1), the identity could be suggested. Finally, a metabolomics approach was carried out with LC–MS data aiming to identify those metabolites capable of discriminating between TT and NP samples.

**TABLE 1:** Retention time and elemental compositions of protonated molecules [M + H]^+^ of acephate and its metabolites with resulting diagnostic product ions using high‐resolution MS/MS experiments.

Nomenclature	Retention time (min)	Molecular formula	Molecular ion mass [M + H]^+^	Molecular ion error (ppm)	Product ions (*m*/*z*)	Product ions error (ppm)
Acephate	1.16	C_4_H_10_NO_3_PS	184.01925	0.073	86.09697	
					125.02119	2.741
					142.99274	0.112
					157.00224	−6.038
O,O,S‐trimethylphosphorothioate	1.16	C_3_H_9_O_3_PS	157.00835	0.032	142.99274	0.112
O,S‐dimethylphosphorothioate	1.16	C_2_H_7_O_3_PS	142.99269	0.078	113.00014	0.248
					128.97721	0.152
Methamidophos	0.96	C_2_H_8_NO_2_PS	142.00869	0.046	113.00007	0.248
					128.97713	0.152
Monomethylphosphate	1.16	CH_5_O_4_P	112.99982	0.258	67.05493	0.703
					95.04963	0.489
Phosphoric acid	0.59	H_3_PO_4_	98.98417	0.448		
Dimethyldisulfide	1.15	C_2_H_6_S_2_	94.99836	0.002		
Acetic acid	0.65	C_2_H_4_O_2_	61.02918	0.720		
Acetamide	0.53	C_2_H_5_NO	60.04439	0.740		

### 3.3 Metabolomics Approach

The LC–MS data obtained for TT and NT samples were submitted in the MS‐DIAL software. Subsequently, the dataset was submitted to an unsupervised and supervised multivariate statistical analysis, PCA, and OPLS‐DA, respectively. The PCA model comprised 80.5% of dataset information, allowing a differentiation among the two clusters, organizing in the same cluster those samples with chemical similarities, while samples chemically distinct were displayed in different clusters. In this way, TT samples were clustered together, in the same group, whereas NT samples were in another group (Figure S3).

Besides, it was crucial to identify the important metabolites for this differential clustering. Therefore, OPLS‐DA was capable of maximizing clustering and separating samples according to acephate treatment. The OPLS‐DA model showed R²Y 0.754 and good cumulative predictive capacity (*Q* ^2^ = 0.98605), as well as, provided the VIPs. Each variable had its fold change and *p* calculated. Herein, it was possible to indicate 3 variables with VIP value > 1, fold change > 2 and *p* < 0.05 (Table 2). Therefore, the metabolomics analysis pointed out the following metabolites to discriminate TT samples from NT samples: monomethylphosphate, O,S‐dimethylphosphorothioate, and O,O,S‐trimethylphosphorothioate (Table 2).

**TABLE 2:** Variables (metabolites) capable to discriminate TT samples from NT samples.

ID	Metabolites	VIP	*p*	Fold change
1436	Monomethylphosphate	1	2.03E‐09	2550.17
2061	O,S‐dimethylphosphorothioate	3	2.19E‐10	2858.61
2352	O,O,S‐trimethyl phosphorothioate	1	1.29E‐09	2414.35

### 3.4 Behavior of Acephate Metabolite Formation

The behavior of acephate biotransformation was evaluated by calculating its concentration for a period of 0–168 h (Figure 1A), as well as by verifying the amount of the formed metabolites through calculating the ratio between the area of the chromatographic peak of the analyte and the internal standard (Figure 1B,C).

Thus, it is possible to observe that acephate concentration decreases quickly in the first 12‐h exposure, and it continues decreasing up to 48‐h exposure, but after 48 h, the acephate concentration remains constant (Figure 1A). Likewise, as it was expected by metabolomics analysis, the metabolites monomethylphosphate, O,S‐dimethylphosphorothioate, and O,O,S‐trimethylphosphorothioate were the main acephate metabolites, as they were generated in higher amounts than the others, and they could be detected since the first hours (Figure 1B,C).

It is important to consider that these 3 metabolites were generated from 0 h to 168 h. However, the metabolites acetamide, acetic acid, dimethyldisulfide, methamidophos, and phosphoric acid were formed in lower amounts (Figure 1C), suggesting that they are less representative of acephate biotransformation made by zebrafish.

**FIGURE 4:**
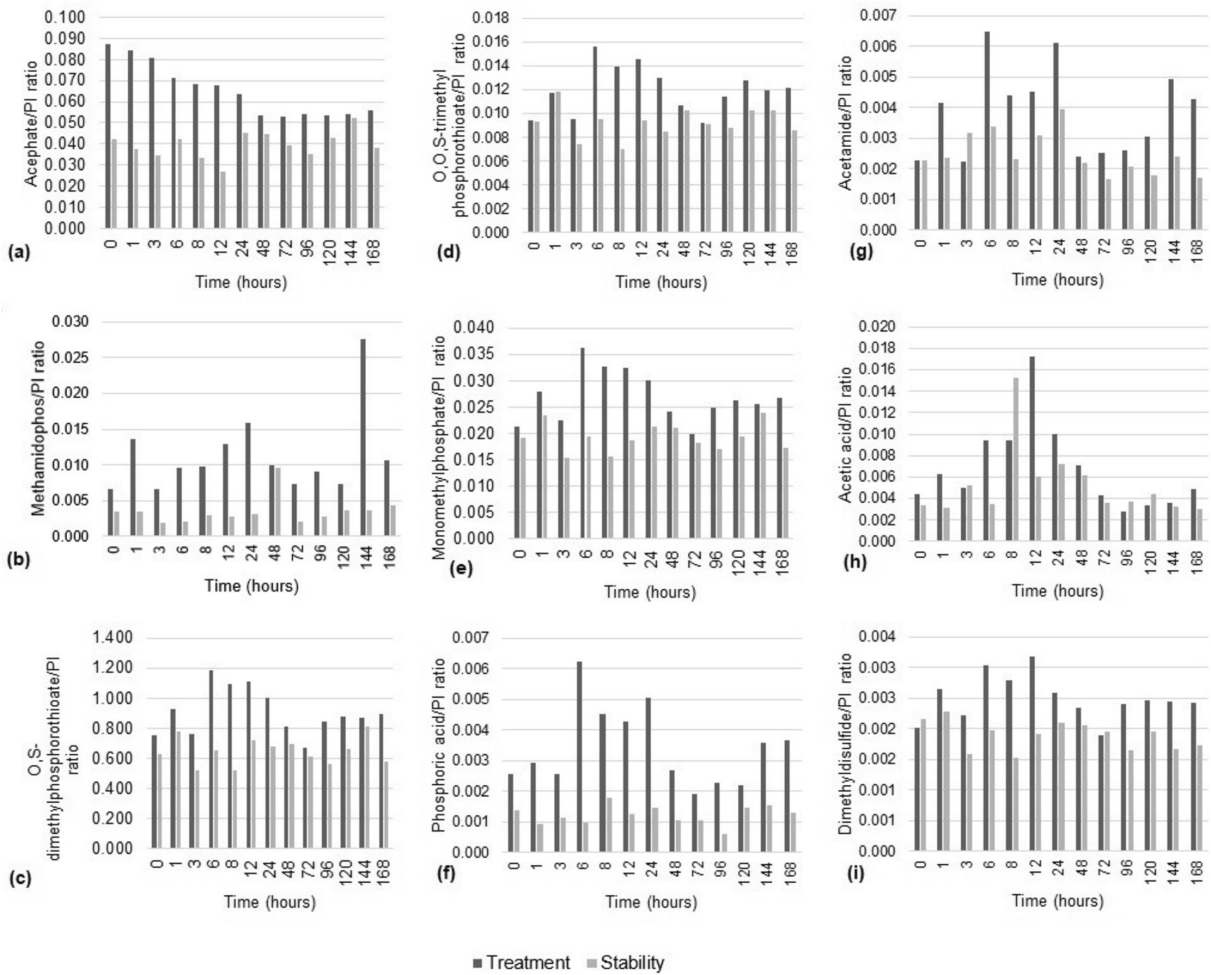
Metabolites generated during the 168 h of acephate exposure according to the ratio between the chromatographic peak area for each metabolite and the internal standard (PI) chromatographic peak area, both extracted from data obtained for the treatment and stability groups.

The acephate metabolites were also detected in ST samples (Figure 4), showing that they could be generated by degradation with no need for fish biotransformation. However, the amount of the metabolites in TT samples was higher than in ST samples, suggesting that the acephate biotransformation by zebrafish showed a great contribution to the amount of generated metabolites.

## 4 Discussion

Methamidophos is the main product of acephate (Figure S2), however according to metabolomics analysis, it was not capable of satisfactorily discriminating between TT and NT as its VIP value was 0.9. Possibly this occurred due to methamidophos being highly generated both by degradation in the environment and by biotransformation occurring in the organisms exposed to it. Another relevant point is the fact that methamidophos gives rise to three other products, as follows: O,S‐dimethylphosphorothioate; O,O,S‐trimethylphosphorothioate; and methyl mercaptan (Lin et al. 2022). It may contribute to a decrease in methamidophos concentration in TT samples that might result in a lack of methamidophos capacity to discriminate between TT and NT clusters.

As far as we know, there are no studies about acephate biotransformation by fish. On the other hand, some studies have reported bacterial degradation of acephate (Lin et al. 2022; Singh et al. 2020; Wu et al. 2023), and a single study has reported the acephate metabolites resulting from mouse biotransformation (Chukwudebe et al. 1984). According to these previous studies, methamidophos is formed by acephate N‐deacetylation (Ren et al. 2020). After that, the P–N bond of methamidophos is cleaved to form O,S‐dimethylphosphorothioate (Wu et al. 2023). Subsequently, O,S‐dimethylphosphorothioate is hydrolyzed at the P–O bond to release ‐OCH3, resulting in O,O,S‐trimethylphosphorothioate (Ren et al. 2020; Wu et al. 2023). Additionally, hydrolysis and dealkylation of O,S‐dimethylphosphorothioate and O,O,S‐trimethylphosphorothioate generate monomethylphosphate (Ren et al. 2020). Therefore, the main reactions involved in acephate biotransformation by the reported organisms (Chukwudebe et al. 1984; Lin et al. 2022; Ren et al. 2020; Singh et al. 2020; Wu et al. 2023) are mainly oxidation, hydrolysis, alkylation, and dealkylation.

It is important to observe that O,S‐dimethylphosphorothioate might be formed from both acephate and methamidophos biotransformation (Lin et al. 2022). It might explain the higher VIP and fold change for O,S‐dimethylphosphorothioate regarding O,O,S‐trimethylphosphorothioate and monomethylphosphate. In other words, it might contribute to the greater role of O,S‐dimethylphosphorothioate in differentiating between TT and NT clusters.

## 5 Conclusions

Acephate is one of the most sold pesticides in Brazil, and despite being banned in many countries, acephate and its main by‐product have been detected in surface and drinking water around the world. In this regard, it is necessary to monitor acephate exposure in aquatic environments, and for that, the knowledge of its biotransformation products is essential.

Herein, we putatively identified the target acephate metabolites generated by zebrafish in a water tank during 168 h of exposure. It is worth noting that the metabolites were identified based on high‐resolution mass spectrometry data and not by comparison with analytical standards. Despite this limitation, the evaluation of acephate metabolites generated in the water tank showed the possibility of using water as a matrix for detecting these metabolites. Besides, the application of a metabolomics approach showed monomethylphosphate, O,S‐dimethylphosphorothioate, and O,O,S‐trimethylphosphorothioate as the most important metabolites for determining acephate exposure. Therefore, the exposure to acephate in aquatic environments could be assessed by monitoring these metabolites, ensuring detectability and high confidence.

## Conflicts of Interest

The authors declare no conflicts of interest.

## Supporting information


**Figure S1:** Acephate—chromatogram (a), mass spectrum in full‐scan (b) and full‐scan DDA‐MS2 (c). Methamidophos—chromatogram (d), mass spectrum in full‐scan (e) and full‐scan DDA‐MS2 (f).

**Figure S2:** Molecular structure of acephate and its metabolites.

**Figure S3:** PCA score plot obtained for TT (tanks treated with acephate) and NT (negative tank) samples.

## Data Availability

The data used in this study are available on the Mendeley Data, V1, doi: 10.17632/srpvvm4wcw.1.
