# Supplementary figures and images for: Evaluation of Acephate Metabolites Generated by Zebrafish ( Danio rerio ) Using LC–HRMS and Metabolomics Approach
Source: J Appl Toxicol. 2025 Nov 17;46(5):1679–86. doi: 10.1002/jat.4988 (PMC13040439; doi:10.1002/jat.4988)

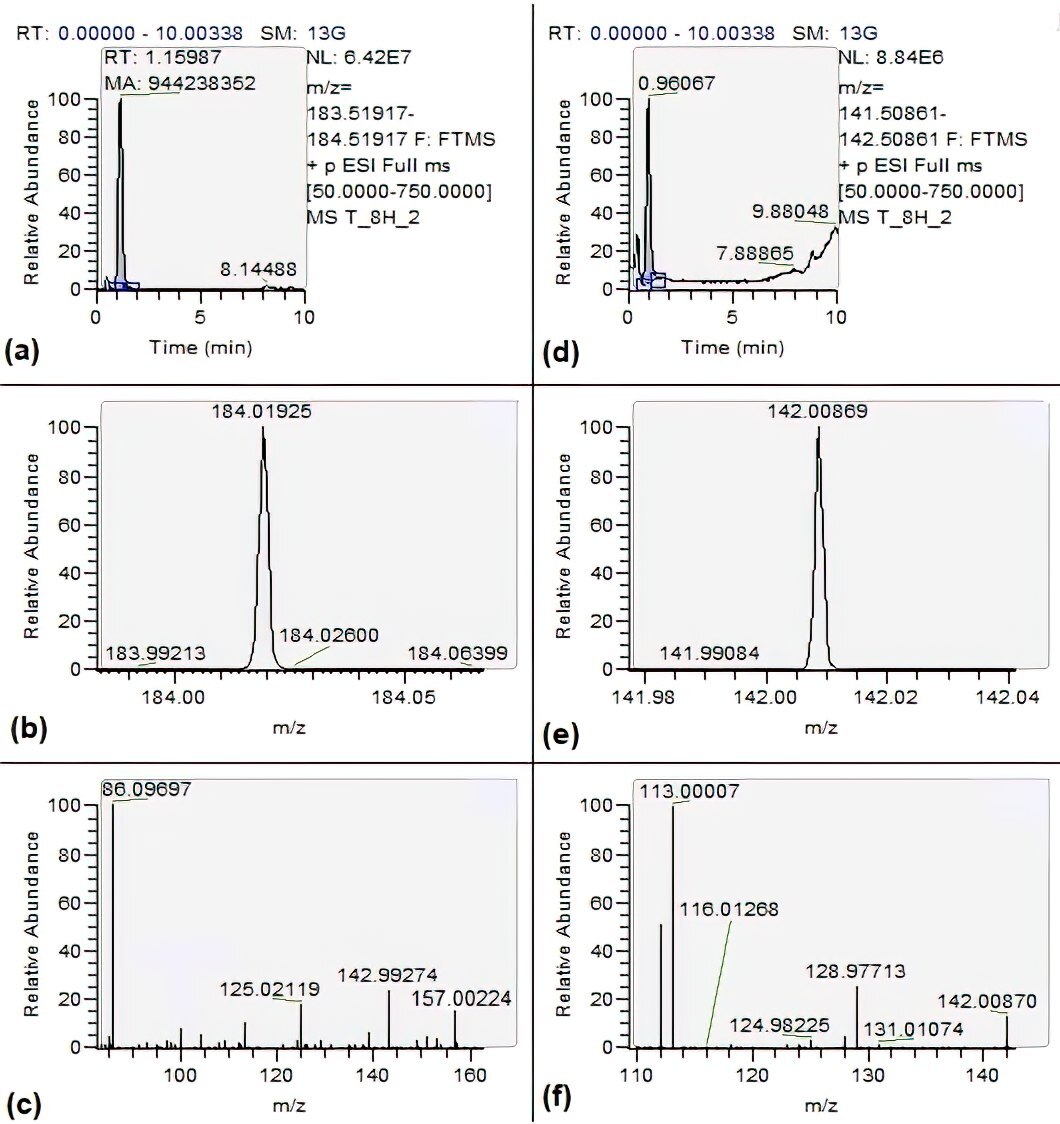

Supplement: Supplementary file 1 — Figure S1: Acephate—chromatogram (a), mass spectrum in full‐scan (b) and full‐scan DDA‐MS2 (c). Methamidophos—chromatogram (d), mass spectrum in full‐scan (e) and full‐scan DDA‐MS2 (f). [file JAT-46-1679-s001.jpeg]

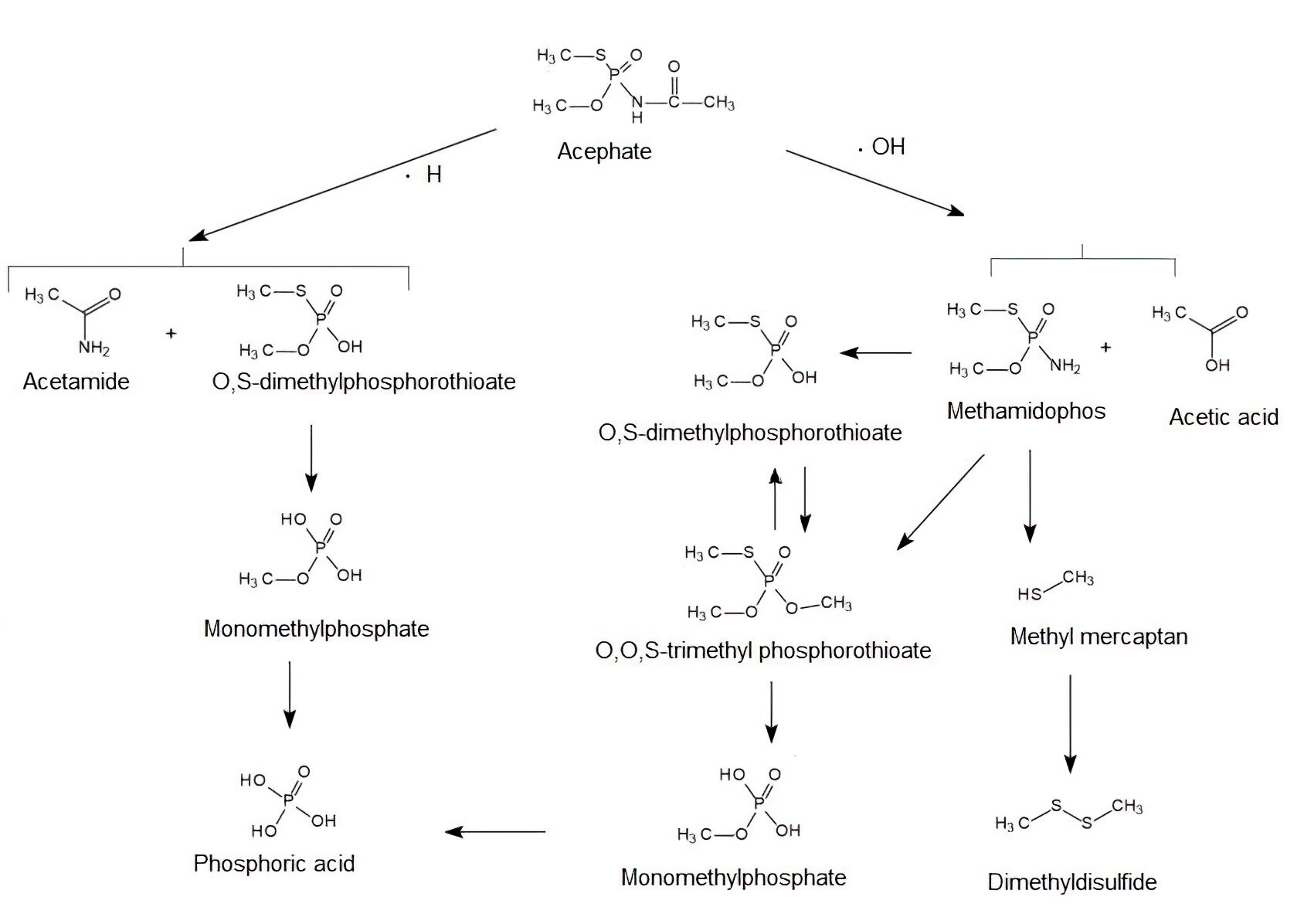

Supplement: Supplementary file 2 — Figure S2: Molecular structure of acephate and its metabolites. [file JAT-46-1679-s003.jpeg]

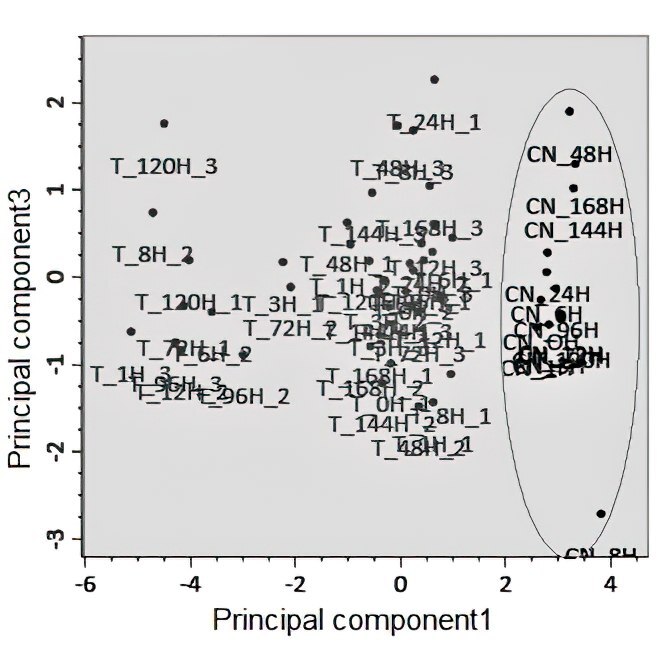

Supplement: Supplementary file 3 — Figure S3: PCA score plot obtained for TT (tanks treated with acephate) and NT (negative tank) samples. [file JAT-46-1679-s002.jpeg]
